# *Scuticeratina*: A new subgenus of small carpenter bees (Hymenoptera, Apidae, Xylocopinae) from Indomalaya

**DOI:** 10.3897/zookeys.1269.148092

**Published:** 2026-02-13

**Authors:** Trevor J. L. Sless, Erika M. Tucker, Sandra M. Rehan

**Affiliations:** 1 Department of Biology, York University, Toronto, Ontario, Canada Milwaukee Public Museum Milwaukee United States of America; 2 Milwaukee Public Museum, WI, Milwaukee 53233, USA Department of Biology, York University Toronto Canada; 3 Biodiversity Outreach Network, AZ, Arizona 86001, USA Biodiversity Outreach Network Arizona United States of America

**Keywords:** *
Ceratina
*, new species, small carpenter bees, systematics, taxonomy

## Abstract

Small carpenter bees (*Ceratina*) are a morphologically diverse group of bees with a cosmopolitan distribution. The ~370 species of this group have been further classified in 25 different recognized subgenera. Here, a distinct new subgenus from Indomalaya, *Scuticeratina***subgen. nov**., with two new species, *Ceratina ferruginea***sp. nov**. and *Ceratina phukhaensis***sp. nov**., are described. Two described species of previously uncertain subgeneric status, *C. muscatella* Nurse, 1902 **stat. rev**. and *C. splendida* Shiokawa, 2008 **stat. rev**. are also included within *Scuticeratina*. The monophyly of this group is supported by both morphological characters and molecular data, including phylogenetic analyses based on targeted genes and ultraconserved elements. Additionally, a third new species is tentatively placed in the nominate subgenus *Ceratina* Latreille, 1802, *C. sirikitae***sp. nov**., distinct from other known species of this subgenus with unusual red abdominal coloration. Finally, an updated key to the *Ceratina* subgenera of the eastern hemisphere incorporating the new subgenus is provided.

## Introduction

The genus *Ceratina* Latreille, 1802 (Hymenoptera: Apidae: Xylocopinae) comprises a cosmopolitan group of species commonly known as small carpenter bees (Michener 2007; Ascher and Pickering 2024). In addition to their already extensive distribution, both historical and recent studies have documented additional range expansions by some species (likely via accidental human transport of nests in plant material) that may require monitoring to ensure they do not negatively affect native bee species (Daly 1966; Groom et al. 2017; Shell and Rehan 2019). *Ceratina* species, as generalists, pollinate a variety of flowers and carve their nests out of dead twigs or stems that have pithy cores (Daly 1973; Mitchell 1962; Michener 2007). The genus also includes a range of social behaviors, with some solitary species as well as many that exhibit subsocial or facultatively social behavior (Sakagami and Maeta 1977; Michener 2007; Rehan et al. 2009, 2010, 2014; Sless and Rehan 2023).

*Ceratina* contains ~370 described species (Ascher and Pickering 2024), representing a broad range of morphological diversity, which has contributed to the establishment of 25 currently recognized subgenera (Michener 2007; Terzo et al. 2007; Warrit et al. 2012; Roig-Alsina 2016; Engel 2023). Most *Ceratina* diversity is found in Africa, Eurasia, and Australasia, with 20 of the known subgenera and 64% of species occurring in the Eastern Hemisphere (Ascher and Pickering 2024). Recent work has revealed further diversity in this genus represented by morphologically challenging or cryptic species (Shiokawa 2008, 2009; Rehan and Richards 2010; Rehan and Sheffield 2011; Flórez-Gómez et al. 2022). There have been a number of detailed morphological and molecular studies on subsets of *Ceratina* (van der Vecht 1952; Yasumatsu and Hirashima 1969; Hirashima 1971; Eardley and Daly 2007; Warrit 2007; Shiokawa 2008, 2009, 2015; Rehan et al. 2010; Rehan and Schwarz 2015; Sless et al. 2024), but comprehensive revision of the whole group is still much needed. Our research contributes novel information on the systematics and identification of bees in the genus *Ceratina*. Here, we propose a distinct new subgenus of small carpenter bees, describe three new species, and provide an updated key to *Ceratina* subgenera of the Eastern Hemisphere.

## Materials and methods

### Taxon and gene sampling

This study included DNA sequence data available in GenBank from Rehan and Schwarz (2015) and Shell and Rehan (2019), in addition to newly generated sequence data. Previously published sequences from three genes were complemented with the addition of corresponding sequences from seven additional specimens (Table 1). The final dataset included 117 *Ceratina* representing 18 subgenera from around the world, as well as 25 outgroup taxa from other genera of Apidae. Newly included sequences represented one previously described species, *Ceratina dentipes kankauensis* (Strand, 1913), and three new species, two of them belonging to the subgenus *Scuticeratina* subgen. nov. Vouchers and type specimens are deposited in the Rehan Collection at York University (RCYU). Additionally, ultraconserved element (UCE) sequence data from one specimen each of *Ceratina ferruginea* sp. nov. and *C. sirikitae* sp. nov. was combined with UCE contigs from 185 specimens previously included in the UCE phylogeny of Sless et al. (2024) to further support the position of these new taxa with respect to other subgenera.

**Table 1.** GenBank accession numbers for new specimens included in the molecular analysis.

**Species**	**Voucher ID**	**COI**	**CytB**
C. (Neoceratina) dentipes kankauensis	Thai 29	MH118559	MH118566
C. (Neoceratina) dentipes kankauensis nr.	Thai 23	MH118565	MH118567
C. (Ceratina) sirikitae sp. nov.	rs2Tha01	MH118561	MH118568
C. (Ceratina) sirikitae sp. nov.	rs2Tha02	MH118562	MH118569
C. (Ceratina) sirikitae sp. nov.	rsTha03	MH118563	MH118572
C. (Scuticeratina) phukhaensis sp. nov.	S05Tha01	MH118564	MH118570
C. (Scuticeratina) ferruginea sp. nov.	rs1Tha01	MH118560	MH118571

### Molecular data

One nuclear gene region, the F2 paralogue of elongation factor-1 alpha (EF-1α F2; 770 bp), as well as the mitochondrial gene regions cytochrome c oxidase subunit I (COI; 1283 bp) and cytochrome b (CytB; 432 bp), were targeted for acquisition (Rehan et al. 2010; Rehan and Schwarz 2015). DNA tissue sampling, extraction, PCR amplification, and sequencing followed the methods in Rehan et al. (2010) for EF-1α and CytB, and Shell and Rehan (2019) for COI. New sequences for each gene region were aligned with previously published sequence data using MAFFT (Katoh and Standley 2013) and then concatenated in Mesquite v. 4.0 (Maddison and Maddison 2025) to create a final alignment of 2485 bp. GenBank accession numbers for newly generated sequence data are provided in Table 1. To further improve the taxon sampling of this three-gene tree, we also used BLAST v. 2.17 (Camacho et al. 2009) to extract COI, CytB, and EF-1α sequences from selected previously published UCE contigs for species of interest as likely sister groups to our newly described taxa. UCE sequence data for *C. ferruginea* and *C. sirikitae* were generated alongside other specimens used in Sless et al. (2024) following previously established protocols (Faircloth et al. 2012; Branstetter et al. 2017, 2021; Grab et al. 2019).

### Phylogenetic reconstruction

The three-gene alignment was partitioned by locus and codon position, with partitions merged and models selected using ModelFinder2 (Kalyaanamoorthy et al. 2017) as implemented in IQ-Tree v. 2.3.6 (Chernomor et al. 2016; Minh et al. 2020). We specified the use of codon substitution models and constrained relationships following the topology of higher-level clades “A–F” in Sless et al. (2024), though did not constrain the positions of the newly described species herein. In parallel, we also generated a UCE alignment produced using the same approach described in Sless et al. (2024). Briefly, UCE loci were identified and extracted from contigs using the Phyluce v. 1.7.1 package (Faircloth 2016), then aligned with MAFFT (Katoh and Standley 2013) and trimmed with Gblocks (Talavera and Castresana 2007) and Spruceup (Borowiec 2019). The alignment was filtered for 75% taxon occupancy in Phyluce and partitions were created by splitting each UCE locus into left, core, and right regions based on entropy using the sliding window site characteristics approach (SWSC-EN; Tagliacollo and Lanfear 2018) before merging partitions as described above. Tree searches were conducted with 1000 replicates for both ultrafast bootstraps (Hoang et al. 2018) and SH-like approximate likelihood ratio testing (Guindon et al. 2010) to assess branch support. Trees were visualized and pruned for clarity using FigTree v. 1.4.4 (Rambaut 2012) as well as the phytools package in R v. 4.3.2 (Revell 2012; R Core Team 2023). Genetic divergence between species for each gene was estimated using Mesquite v. 4.0 (Maddison and Maddison 2025) with the Kimura 2-parameter substitution model.

### Morphology and specimens

Morphological terminology largely follows that used in Michener (2007) and Shiokawa (2009). Images of bee specimens were taken using a Canon EOS 7D camera with 65 mm macro lens and the software EOS Utility2 v. 2.14.10.2 and Adobe Lightroom v. 14.4. Multiple image layers were then stacked with Zerene Stacker (v. 1.04) and edited with Adobe Photoshop CC 2015.5.0. The ‘Key to the Eastern Hemisphere Subgenera of *Ceratina*’ is reproduced from Michener (2007) with slight modifications to incorporate the new subgenus and new species. Type and non-type specimens for comparison of morphological features of new subgenera and species were loaned from the following institutions:

**BPBM** Bernice P. Bishop Museum, Honolulu, Hawaii, USA

**ELKU** Entomological Laboratory, Faculty of Agriculture, Kyusyu University, Fukuoka, Japan

**SEHU** Hokkaido University Museum, Sapporo, Japan

**ZMHB** Museum für Naturkunde der Humboldt-Universität, Berlin, Germany

**NMNS** National Museum of Natural Science, Taichung, Taiwan

**RCYU** Rehan Collection, York University, Toronto, Canada

**SDEI** Senckenberg Deutsches Entomologisches Institut, Müncheberg, Germany

**OUMNH** University Museum of Natural History, Oxford, United Kingdom

## Results

### Phylogenetic reconstruction

**Figure 1.**
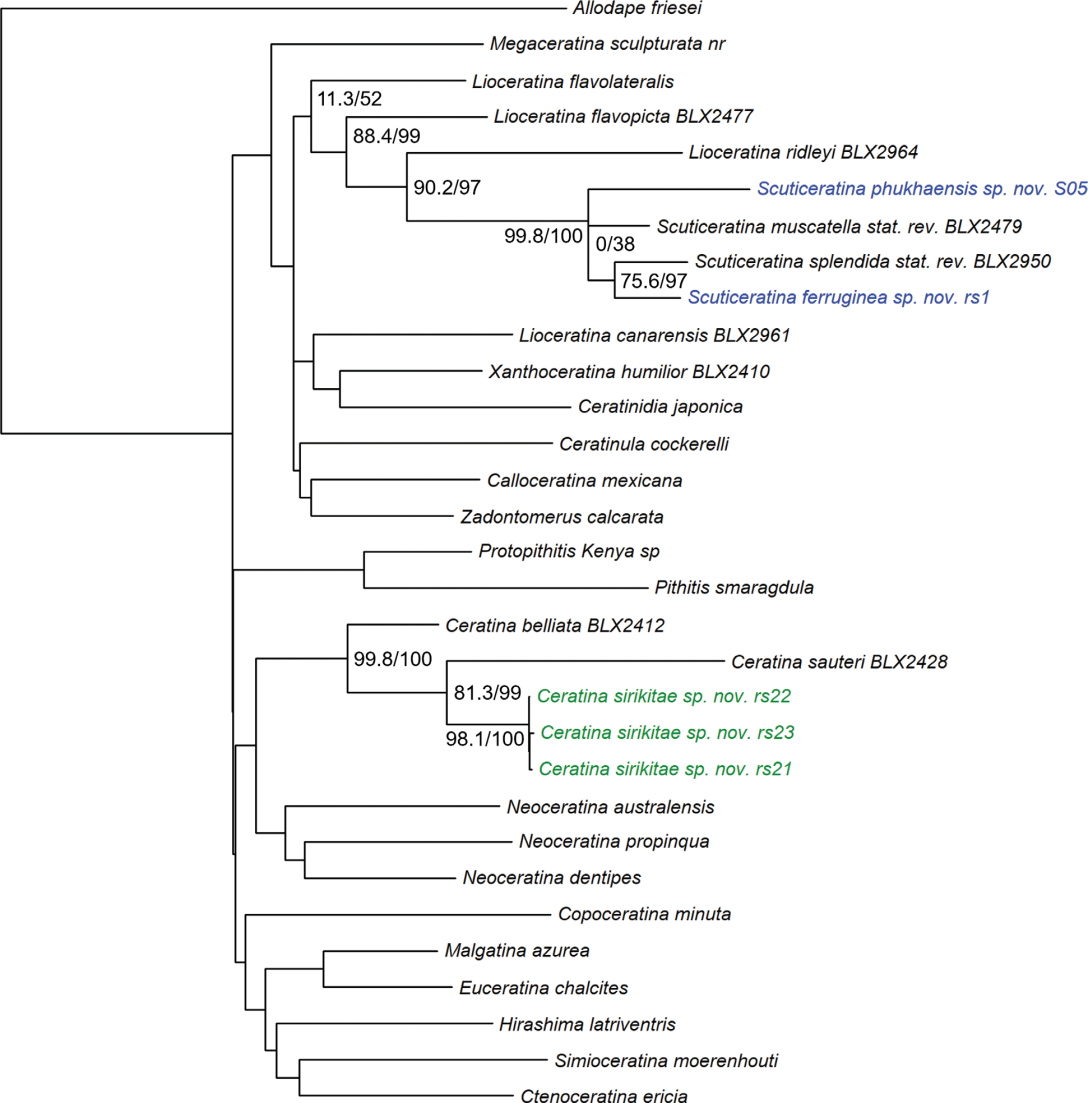
IQ-tree three-gene phylogeny pruned to highlight major *Ceratina* subgenera and placement of new species. Specimens of the new subgenus *Scuticeratina* are in blue and specimens of the new species C. (Ceratina) sirikitae are in green. Support values at indicated nodes represent ultrafast bootstrap and SH-alrt values respectively; see Suppl. material 1 for all support values.

**Figure 2.**
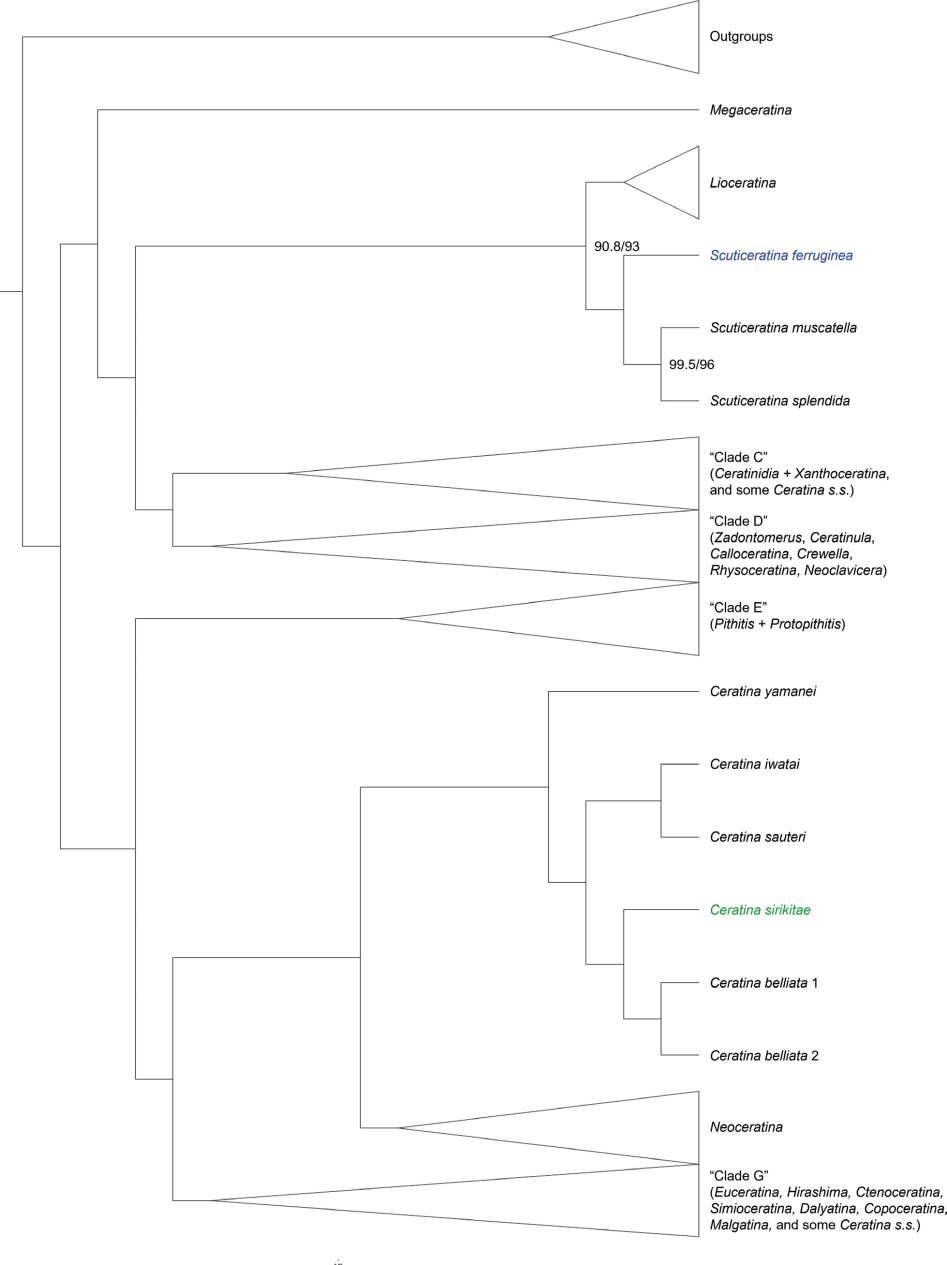
IQ-tree UCE phylogeny with major clades collapsed. The new species Ceratina (Scuticeratina) ferruginea and C. (Ceratina) sirikitae are in blue and green, respectively. Letters for some collapsed groups refer to equivalent clades in Sless et al. (2024). Support values at indicated node represent ultrafast bootstrap and SH-alrt values respectively; all other nodes shown had 100/100 support; see Suppl. material 2 for all support values.

The phylogeny based on the three-gene dataset recovered three new molecularly distinct taxa. *Ceratina phukhaensis* sp. nov. and *C. ferruginea* sp. nov. form a well-supported monophyletic group with *C. muscatella* and *C. splendida* that is morphologically diagnosable and distinct from other subgenera, and is hence proposed as a new subgenus (ultrafast bootstrap/SH-alrt branch support 99.8/100; Fig. 1, Suppl. material 1). The position of these species in the UCE tree suggests that the closest relatives of *Scuticeratina* are within subgenus *Lioceratina* Vecht, 1952 (branch support 90.8/93; Fig. 2, Suppl. material 2). This is consistent with the close relationship with *Lioceratina* recovered in the three-gene phylogeny, although *Lioceratina* is recovered as paraphyletic in both our trees. Genetic divergence between the specimen of C. (Scuticeratina) phukhaensis and C. (Scuticeratina) ferruginea (COI: 11.34% divergence, Suppl. material 3: table SS1; CytB: 10.43% divergence, Suppl. material 3: table S2) was comparable to the divergence range seen between different species within other subgenera (Suppl. material 3: tables S1, S2).

All three specimens of the third new species, *Ceratina sirikitae*, formed a tight cluster (branch support 98.1/100) in the three-gene phylogeny, recovered as the sister to *C. sauteri* Strand, 1913 and close to the clade corresponding to subgenus *Neoceratina* Perkins, 1912 (Fig. 1, Suppl. material 1). In the UCE phylogeny, however, *C. sirikitae* was instead recovered as the sister to *C. belliata* Shiokawa, 2008 (branch support 100/100; Fig. 2, Suppl. material 2). *C. belliata* and sauteri are both currently classified under the nominate subgenus *Ceratinasensu stricto*, although in the full UCE phylogeny of Sless et al. (2024) these species form the sister group to *Neoceratina* along with C. (Ceratina) iwatai Yasumatsu, 1936 and C. (C.) yamanei Sung & Shiokawa, 2012 rather than grouping with other members of the nominate subgenus. Genetic divergence among the three sequenced specimens of C. (Ceratina) sirikitae was very low (COI: 0.21–0.63% divergence, Suppl. material 3: table SS1; CytB: 0.77–1.03% divergence Suppl. material 3: table S2) compared to that of divergence between the related species *C. belliata* and sauteri (COI: 7.95–8.85% divergence, Suppl. material 3: table SS1; CytB: 9.98–10.56% divergence, Suppl. material 3: table S2) adding support for recognition of the new species.

### Morphological analysis

Diagnostic characters for the new subgenus and species were identified based on a comparative morphological analysis. These characters are given in full in the subgeneric and species descriptions following the key. A key to the subgenera of *Ceratina*, including *Scuticeratina*, for the eastern hemisphere is provided below.

### Key to the Eastern Hemisphere subgenera of *Ceratina* (modified from Michener 2007)

Couplet 4 has been rewritten to separate the subsequent groups of subgenera more clearly and account for exceptions to some features. Couplet 7 is newly added to separate *Scuticeratina* and *Catoceratina* Vecht, 1952 with some slight corresponding changes to couplet 6. All other couplets remain unmodified aside from updating their numbering, formatting, and geographical terminology.

We have been unable to examine material from the recently described subgenera *Xestoceratina* Engel, 2023 and *Alloceratina* Engel, 2023, and as such these are not included in the key. As discussed in their original diagnoses, however, *Xestoceratina* should key to *Copoceratina*, while *Alloceratina* should key to either *Ceratina* s.s. or couplet 15 (Engel 2023).

**Table:** 

1	Prestigma approx. as long as distance from base of stigma to vein r; stigma not wider than prestigma, as measured to wing margin; margins of stigma basal to vein r parallel, margin in marginal cell not convex; distal halves of middle and hind femora with sharp, bladelike edges ventrally (Africa)	**Ceratina (Megaceratina)**
–	Prestigma much shorter than distance from base of stigma to vein r; stigma wider than prestigma, as measured to wing margin (except in large species like *Ceratina chalcites* Germar); margins of stigma basal to vein r convergent basally, margin in marginal cell convex except in largest species; middle and hind femora without sharp ventral edges	**2**
2(1)	T2–T6 without graduli (punctation very strong; punctures of lower part of paraocular area usually large, as close as they can be, flat bottomed so that the pattern is a network of ridges; supraclypeal area with transverse carina below antennae; basal area of propodeum not longer than metanotum; notaulus extending nearly to posterior margin of scutum; hind tibia of female with no evidence of basitibial plate)	**3**
–	At least T2 and T3, and sometimes subsequent terga, with graduli	**4**
3(2)	Axilla produced to posterior angle separated from scutellum; basal area of propodeum separated from posterior surface by carina; T7 of male rounded or pointed (Africa, Indomalaya)	**Ceratina (Pithitis)**
–	Axilla not angulate, abutting scutellum; basal area and posterior surface of propodeum not separated by carina; T7 of male bidentate (Africa)	**Ceratina (Protopithitis)**
4(2)	Frons above antennal socket nearly or completely impunctate, though sometimes with a few fine punctures along inner margin of eye; paraocular area and usually genal area with pale marking in both sexes; body usually with yellow, ferruginous, or rarely red; when present, yellow or red coloration extending to basal pregradular areas of terga (except in C. (Catoceratina) perforatrix, which can be distinguished from *Ceratinidia* by the 2^nd^ tergite lacking any yellow in females or with very small yellow marks in males)	**5**
–	Frons above antennal socket punctate, usually strongly so; or if this area is impunctate, then body small, with pale markings restricted to head, pronotal lobe, and legs or entirely absent (some members of *Neoceratina* and *Ceratina* s.s.); paraocular areas without yellow markings in female (except for *Ceratinidia*); pregradular areas black or rarely red/ferruginous, yellow coloration on metasoma, if present, restricted to apical portions of terga	**9**
5(4)	Basal area of propodeum horizontal, sharply separated from posterior surface, which is steeply declivous; antenna of male long, third segment of flagellum longer than broad (apex of T7 of male bluntly tridentate, the median tooth large, triangular; S6 of male with large concavity in middle of subapical portion; head and thorax largely impunctate; dark parts of body slightly to distinctly metallic blue or green (Philippines)	**Ceratina (Chloroceratina)**
–	Basal area of propodeum usually strongly slanting, not abruptly separated from posterior surface; antenna of male short, third segment of flagellum broader than long	**6**
6(5)	Preoccipital carina present, especially strong in male, forming angle between vertex and occiput (Indomalaya)	**7**
–	Preoccipital carina absent, vertex rounded onto occiput	**8**
7(6)	Metasoma black with transverse yellow bands on T3–5; female with ventral scopa consisting of dense patches of long silvery hairs on S2–5; T7 of male with apex truncate and bluntly bituberculate	**Ceratina (Catoceratina)**
–	Metasoma black to mostly red, lacking any yellow maculations; female without ventral scopa (hairs on S2–S5 sparse and largely restricted to apical 1/2 of sterna); T7 of male with medial projection, narrowly bidentate at apex	**Ceratina (Scuticeratina)**
8(6)	Basitibial plate of female rudimentary; at least median part of clypeus finely coriaceous in female; basal area of propodeum also finely coriaceous, not coarsely sculptured; genitalia of male without bundles of hairs (Indomalaya)	**Ceratina (Lioceratina)**
–	Basitibial plate of female distinct, although small; clypeus polished, not coriaceous, or rarely finely coriaceous in female as in *Lioceratina*; basal area of propodeum often slightly more coarsely sculptured than in *Lioceratina*; genitalia of male with four bundles of hairs (Indomalaya)	**Ceratina (Xanthoceratina)**
9(4)	Posterior margins of T2–T5 and S2–S5 each with row of coarse, posteriorly directed setae that are usually thickened, sometimes scale like, the rows on terga sometimes interrupted middorsally; graduli limited to T2, T3, S2, S3, and sometimes S6 of male	**10**
–	Terga and sterna without apical rows of specialized setae; graduli present on T2, T3, and usually T4 of female and on T2–T4 of male, also usually present behind S3 but sometimes weak on S4, etc.	**11**
10(9)	Scutellum strongly convex in profile, its posterior part nearly vertical; profile of metanotum and propodeum strongly declivous (Africa)	**Ceratina (Simioceratina)**
–	Scutellum gently convex, its posterior part and metanotum and propodeum forming a single slope as seen in profile (Africa)	**Ceratina (Ctenoceratina)**
11(9)	T5 with distinct gradulus in both sexes	**12**
–	T5 without gradulus (or with gradulus in male of *Copoceratina* which can be recognized by leg characters listed in couplet 17)	**15**
12(11)	Black with yellow markings on head, thorax, metasoma, and legs (frons and vertex densely and rather coarsely punctate; mesopleura densely punctate; medium sized to large, robust species) (Indomalaya, east Palearctic)	**Ceratina (Ceratinidia)**
–	Black or metallic, with only a few pale markings, if any, on head, pronotal lobe, and legs; metasoma without yellow markings	**13**
13(12)	Genal and frontal areas smooth, largely impunctate except on upper part of genal area, which is punctate, sometimes scattered punctures along frontal margin of eye [but see note on *C. parvula* Smith under the subgenus *Ceratina* s.s.]	**Ceratina (Ceratinula)**
–	Genal and frontal areas punctate, at least a row of dense punctures along frontal margin of eye and scattered punctures on most of genal area	**14**
14(13)	Maxillary palpus 5- or 6-segmented; S5 of female with gradulus; T6 of male without gradulus; T7 of male truncate or rounded or pointed posteriorly; S2 of male without tubercle; gonostylus of male without down-curved projection (Palearctic, Africa, Indomalaya)	***Ceratina* (*Ceratina* s.s.)**
–	Maxillary palpus 5-segmented; S5 of female without gradulus; T6 of male usually with gradulus; T7 of male usually extending posteriorly as long projection that is simple or bidentate at apex; S2 of male usually with tubercle in middle; gonostylus of male with down-curved projection (Palearctic, Indomalaya, Australia)	***C* eratina (*Neoceratina* )**
15(11)	Male gonostylus several times as long as broad, simple, hairy, well separated from gonocoxite; T6 with median longitudinal keel; T7 of male strongly extending posteriorly, simple or bidentate at apex (Palearctic)	**Ceratina (Euceratina)**
–	Male gonostylus short, < 2× as long as broad, with one or two pointed processes or hooks and without hairs or with hairs on restricted areas; T6 not keeled; T7 variable, not so extended (Africa or Madagascar)	**16**
16(15)	Body dark metallic blue; posterior femur of male without tooth or comb of hairs (Madagascar)	**Ceratina (Malgatina)**
–	Body nonmetallic black or with weak bronze reflections; posterior femur of male with ventrobasal tooth or with ventral comb of hairs	**17**
17(16)	Posterior femur of male with ventrobasal tooth, without median ventral comb of hairs; T6 of male without median convexity; terga without apical plumose hairs (Africa, Madagascar)	**Ceratina (Copoceratina)**
–	Posterior femur of male without tooth, with median ventral (sometimes interrupted) comb of hairs; T6 of male with large median convexity with punctuation much finer than on nearby areas; T1–T4 (or at least T1 and T2) with apical bands of white plumose hairs laterally (Africa, Madagascar)	**Ceratina (Hirashima)**

### Species descriptions

#### Ceratina (Scuticeratina)

Taxon classificationAnimaliaHymenopteraApidae

Sless, Tucker & Rehan subgen. nov.

0965AFB9-925C-5B7E-936B-63FF43EF1F63

https://zoobank.org/08CD7156-F455-45C0-965A-8994391BBCDB

Figs 3, 4, 5

##### Type species.

C. (Scuticeratina) ferruginea sp. nov. by original designation.

**Figure 3.**
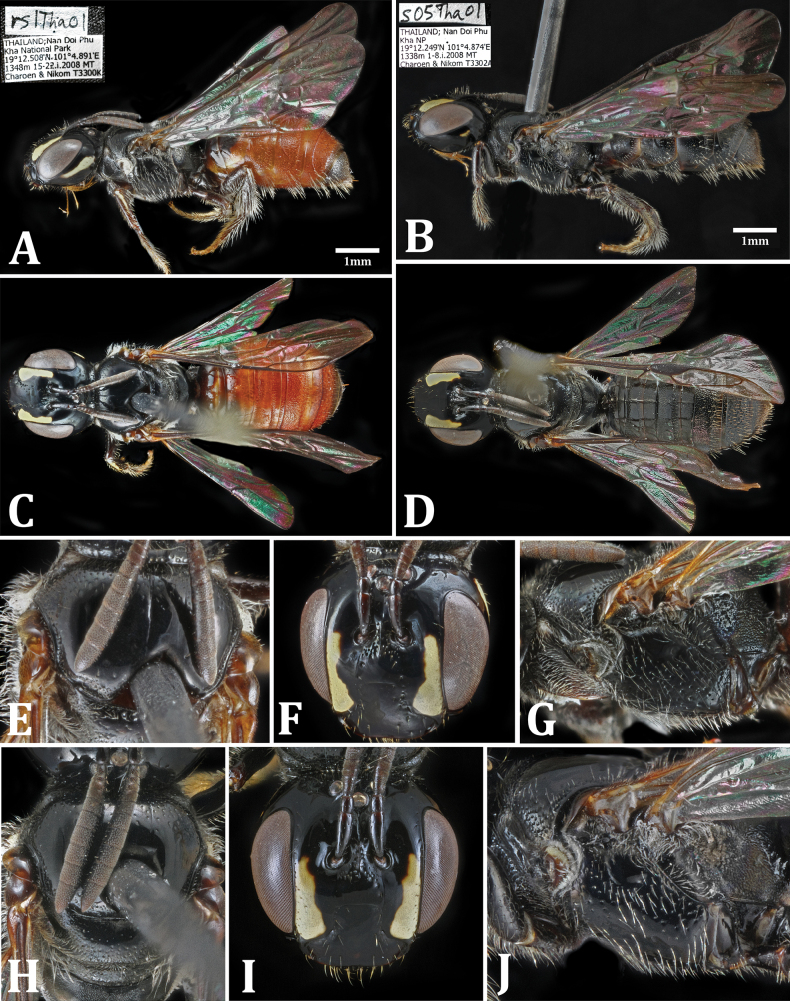
Holotype specimens of newly described *Scuticeratina* species. Ceratina (Scuticeratina) ferruginea sp. nov.: **A**. Lateral habitus; **C**. Dorsal habitus; **E**. Mesoscutum; **F**. Face; **G**. Mesopleuron. Ceratina (Scuticeratina) phukhaensis sp. nov.: **B**. Lateral habitus; **D**. Dorsal habitus; **H**. Mesoscutum; **I**. Face; **J**. Mesopleuron.

**Figure 4.**
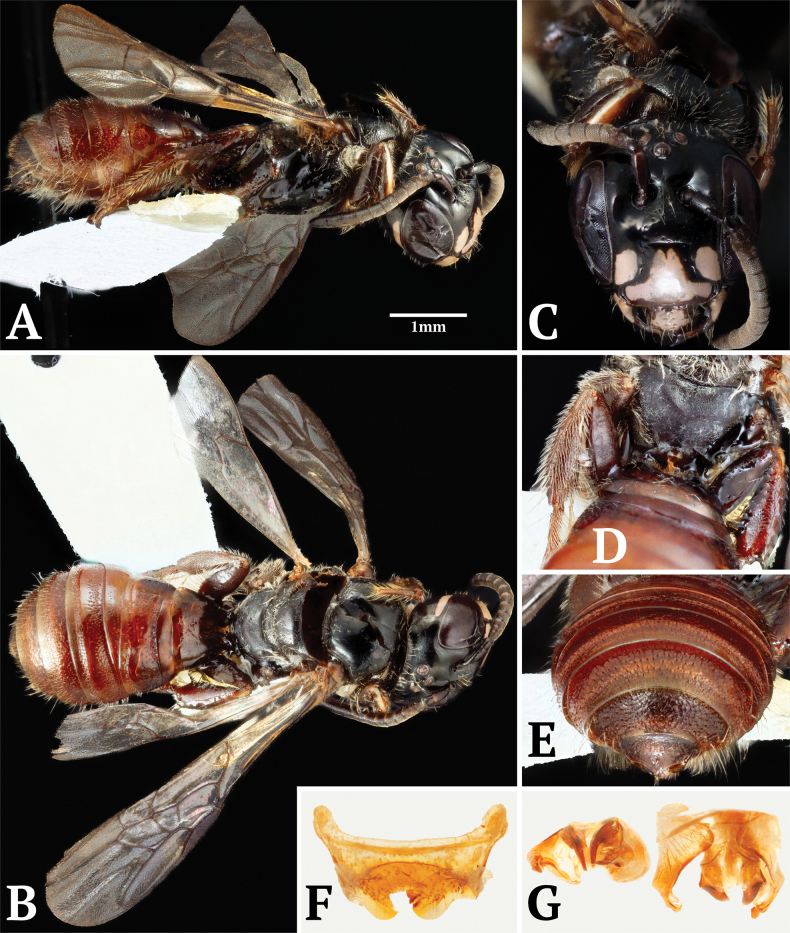
Paratype male specimen of Ceratina (Scuticeratina) ferruginea sp. nov. **A**. Lateral habitus; **B**. Dorsal habitus; **C**. Face; **D**. Propodeum and hindlegs; **E**. Seventh abdominal tergite; **F**. Sixth abdominal sternite; **G**. Genital capsule.

**Figure 5.**
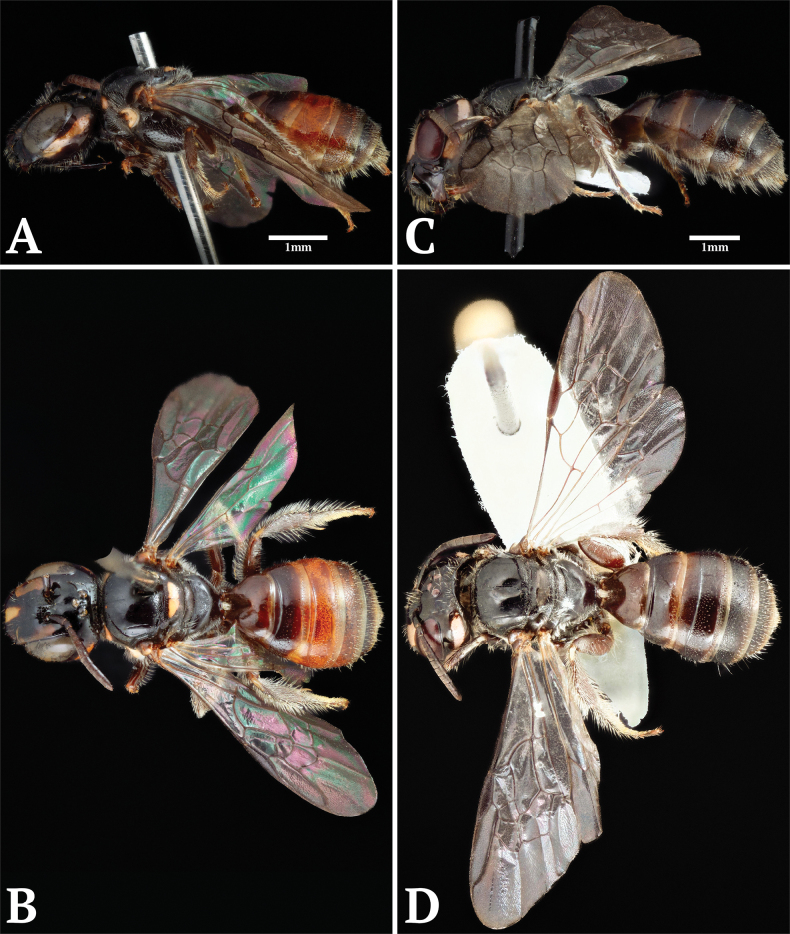
Specimens of previously described species included in *Scuticeratina*. Ceratina (Scuticeratina) muscatella stat. rev.: **A**. Lateral habitus; **B**. Dorsal habitus. Ceratina (Scuticeratina) splendida stat. rev.: **C**. Lateral habitus; **D**. Dorsal habitus.

##### Diagnosis.

The following combination of characters distinguish this subgenus: strong preoccipital carina present; very limited punctation present on the face, especially above antennae; ventral scopa absent; paraocular area with pale maculations in both sexes; abdominal terga lacking yellow maculations (though extensively ferruginous); graduli present on T2–T5 and S2–S5; basitibial plate indistinct. Male T7 pointed and bidentate; S6 with flat apical lobes obscuring small pair of teeth; genitalia without bundles of hair, with tip of gonostylus bifid.

##### Description.

Body length 6–7 mm; forewing 4.5–5 mm; head 1.6–1.88 mm wide × 1.5–1.8 mm long.

***Color***: All shiny, black varying to mostly black with a red abdomen; pale maculations present on paraocular area, gena, and tibiae (sometimes on clypeus, pronotum, and scutellum), but absent from scutum and metasoma.

***Head***: Frons above antennae sparsely punctate; preoccipital carina present.

***Mesosoma***: Scutum largely impunctate; scutellum moderately pitted; metanotum densely pitted; propodeum imbricate to shagreened; propleuron densely pitted; mesopleuron moderately pitted, hypoepimeral area impunctate; metapleuron densely pitted; basitibial plate indistinct.

***Metasoma***: T2–T5 and S2–S5 with graduli; specialized abdominal setae and ventral scopa absent.

##### Distribution.

Thailand, Laos, India, Pakistan, Nepal.

##### Etymology.

Meaning “shield *Ceratina*”, in reference to the strong preoccipital carina exhibited by members of the subgenus. Taken from the Latin *scutum* meaning ‘shield’.

##### Remarks.

This subgenus is morphologically similar to *Catoceratina*, *Lioceratina*, *XanthoceratinaCeratinidia*, and some members of *Ceratinasensu stricto*. It can however be readily distinguished from these subgenera by a number of morphological characteristics. There is a strong preoccipital carina present, which is absent in *Lioceratina*, *Xanthoceratina*, and *Ceratina* s.s. The ventral scopa, which is present in *Catoceratina*, is absent in *Scuticeratina*. The strongly defined basitibial plate in *Catoceratina* and *Xanthoceratina* is also absent in *Scuticeratina*. Additionally, yellow maculations are limited to the head, pronotal lobes, scutellum and legs as opposed to the more extensive maculations present on the scutum and/or terga of most *Catoceratina*, *Lioceratina*, *Xanthoceratina*, and *Ceratinidia*.

#### Ceratina (Scuticeratina) ferruginea

Taxon classificationAnimaliaHymenopteraApidae

Sless, Tucker & Rehan sp. nov.

840EFE88-80B7-5809-B596-A178D1907DA5

https://zoobank.org/CB392658-43DF-43DA-8522-E92B9E5AE72A

Figs 3A, 3C, 3E–G, 4

##### Type material.

***Holotype***: Thailand • 1 ♀; “Nan, Doi Phu Kha National Park”; 19°12.508'N, 101°4.891'E; 1348 m; 15–22 Jan. 2008; [malaise trap]; Charoen & Nikom leg.; T3300k; RCYU “rs1Tha01”. ***Paratypes***: Laos • 2 ♂; “Phongsaly prov., Phongsaly env.”; 21°41–2'N, 102°06–8'E; ~1500 m; 6–17 May 2004, 28 May–20 Jun. 2003; Vít Kubáň leg.; RCYU.

##### Diagnosis.

Distinguished from C. (Scuticeratina) phukhaensis by its red abdomen, strong maculations on the gena and fore tibia, and somewhat sparser punctation on the 2^nd^ tergite and sternite. Distinguished from C. (Scuticeratina) muscatella and C. (S.) splendida by the absence of pale maculations on the clypeus (females) and scutellum (both sexes).

##### Description.

♀ (holotype): body length 7 mm; forewing 5 mm; head 1.88 mm wide × 1.8 mm long. ♂ (paratypes): body length 6–6.5 mm; forewing 4.5–5 mm; head 1.6–1.7 mm wide × 1.5–1.6 mm long.

**Female (Fig. 3A, C, E–G). *Color***: Head and mesosoma shiny black (Fig. 3A, C); mandibles ferruginous medially; clypeus and labrum entirely black; paraocular area with pale yellow maculation filling much of space below antennal sockets, this maculation narrowing and following eye margin up to level of antennal socket, separated from antennal socket by roughly its own width (Fig. 3F); genal area with maculation ~3/4 as long as eye; pronotal collar with faint pale maculations laterally, as well as posterior 1/2 of pronotal lobe; tegulae ferruginous and translucent (Fig. 3E); fore tibia with pale maculation covering ~3/4 of its length; mid tibia with tiny, barely noticeable basal spot; hind tibia with basal maculation covering < 1/4 of its length; tarsi ferruginous; abdomen largely bright red except T1 and S1 ferruginous brown, T2 ferruginous basad of apical depression, T6 and S6 black; rims of terga somewhat translucent.

***Head***: Labrum with fairly dense punctation; clypeus with few very sparse punctures except for line of punctures on each side of upper lobe (Fig. 3F); supraclypeus mostly smooth with few punctures along lateral edges, changing to abruptly punctured between antennal sockets; upper frons between antennal sockets and ocelli nearly impunctate, as well as spaces between lateral ocelli and eyes; inner margin of compound eye with a sparse line of very fine punctures; genal area with a few scattered fine punctures; vertex with strong preoccipital carina lined with punctures along margin, a second line of pits a few diameters anterior of carina; vestiture sparse, absent from most of face; rear margin of vertex and genae with a fringe of medium-length erect white hairs; some similar hairs present between antennal bases, between ocelli, and on mandibles and labrum; a few much shorter hairs on clypeus, along inner eye margins, and on scape.

***Mesosoma***: Scutum moderately punctate in anterior third (1–3 diameters apart) but becoming largely impunctate medially, with just a few punctures along lateral and posterior margins (Fig. 3E); scutellum moderately punctured along margins, becoming sparse centrally; metanotum densely but very shallowly punctured; propodeum with dorsal area imbricate to shagreened; propleuron densely punctured; mesopleuron moderately punctured with spaces of 1–3 puncture widths, hypoepimeral area impunctate (Fig. 3G); metapleuron and lower sides of propodeum moderately to densely punctured; fore and mid femora weakly expanded medially and slightly flattened below; fore and mid tibiae slightly narrower than femora, with curved apical spine; hind femur not expanded, similar in width to hind tibia, basitibial plate indistinct; vestiture sparse dorsally; scutum and scutellum hairless except around edges; metanotum with some long white hairs laterally; propleuron with evenly spaced semi-erect medium-length hairs; mesopleuron with long but sparse white hairs, becoming denser on ventral surface; upper sides of propodeum around spiracle with dense patches of short white hairs; fore and mid legs with a few long white hairs on femur and tibia (slightly denser on the latter), scopal hairs on hind leg longer but not much denser; all three basitarsi with denser and more yellow-brown vestiture in comparison to more basal segments of legs.

***Metasoma***: Graduli present on T2–5 and S2–5 (Fig. 3C); T1 smooth with very sparse shallow punctures; T2 pregradular area finely strigulate and impunctate, postgradular area smooth and sparsely punctured (2–5 diameters apart), narrow transverse line of pits in apical depressed area; T3 pregradular area strigulate with narrow line of pits, postgradular area densely and finely punctured (0.5–1 diameters), though impunctate just before apical depression which contains an additional band of fine and dense punctures; T4 pregradular area moderately punctate, postgradular area very densely punctured (< 1 diameter), pits continuing partway into apical depression which is less sharply defined than on T2 and T3; T5 pregradular area strigulate to densely punctured, postgradular area densely punctured with minute pits continuing partway into apical depression; T6 weakly rugoso-punctate with pits somewhat indistinct; S1 smooth; S2–3 punctures restricted to postgradular area (significantly sparser on S2); S4–S5 pregradular area with transverse band of pits in addition to dense postgradular punctation; S6 punctures dense but indistinct; vestiture sparse on terga, mostly consisting of short light hairs along graduli, though T5 and T6 with denser mix of light and dark hairs; S2–S5 with sparse rows of long, erect, white hairs apically, more uniformly distributed on S6; S2 additionally with moderately dense patch of appressed white hairs immediately posterior to gradulus (wax plate), possibly also present on S3 though less distinct.

**Male (Fig. 4)**. Coloration similar to female except as follows: clypeus largely pale except around edges; labrum and mandible bases entirely covered by pale maculation narrowly outlined with black; paraocular markings shorter, only reaching level of upper clypeal margin rather than antennal bases (Fig. 4C); genal maculations thinner and closer to hind margin of eye than in female; pronotal collar with smaller and fainter maculations; fore tibia with pale marking longer, nearly extending the entire length of the segment; mid and hind tibiae also with slightly more extensive markings than in female, though still small; metasoma largely bright red as in female, though coloration may vary slightly among individuals; punctation and vestiture similar to female across whole body except for absence of hair patches around propodeal spiracles; apex of T7 pointed and obscurely bidentate (Fig. 4E); S6 apically produced into two rounded lobes partially obscuring two small medial teeth (Fig. 4F); genitalia without dense bundles of hair, though some sparse hairs present; tip of gonostylus bifid with curved spine opposing blunt thumb-like projection (Fig. 4G).

##### Etymology.

Named in reference to the conspicuously red abdomen. From the Latin *ferrūgineus* (with feminine inflection) meaning ‘dark red’.

##### Distribution.

Thailand, Laos. Specimens collected from tropical and subtropical moist broadleaf forest biome.

#### Ceratina (Scuticeratina) phukhaensis

Taxon classificationAnimaliaHymenopteraApidae

Sless, Tucker & Rehan sp. nov.

7B8E9347-2FCB-5420-A02E-BA233E36581C

https://zoobank.org/8E553A68-94FD-4BE1-8027-E86EDA30171D

Fig. 3B, D, H –J

##### Type material.

***Holotype***: Thailand • 1 ♀; “Nan, Doi Phu Kha National Park”; 19°12.249'N, 101°4.874'E; 1338 m; 1–8 Jan. 2008; [malaise trap]; Charoen & Nikom leg.; T3302A; RCYU “S05Tha01”.

##### Diagnosis.

Distinguished from C. (Scuticeratina) ferruginea by its entirely black abdomen, reduced or absent maculations on the gena, pronotal collar, and fore tibia, and somewhat denser punctation on the 2^nd^ tergite and sternite. Distinguished from C. (Scuticeratina) muscatella and splendida by the absence of pale maculations on the clypeus (females) and scutellum (both sexes).

##### Description.

**Female (Fig. 3B, D, H–J)**. ♀ (holotype): body length 6.5 mm; forewing 4.5 mm; head 1.7 mm wide × 1.63 mm long.

***Color***: Entire body shiny black (Fig. 3B, D); mandibles, clypeus, and labrum black; paraocular area with pale yellow maculation filling much of space below antennal sockets, this maculation narrowing and following eye margin up to level of antennal socket, separated from antennal socket by slightly more than its own width (Fig. 3I); genal area with small maculation much shorter than eye (1–2 ocellar diameters in length); pronotal collar black, though pronotal lobe with small maculation along posterior edge; tegulae dark brown/testaceous and translucent (Fig. 3H); fore tibia with tiny pale maculation basally, barely detectable on mid and hind tibiae; tarsi dark brown; rims of terga dark brown and slightly translucent.

***Head***: Labrum with fairly dense punctation; clypeus with few very sparse punctures except for line of punctures on each side of upper lobe (Fig. 3I); supraclypeus mostly smooth with few punctures along lateral edges, changing to abruptly punctured between antennal sockets; upper frons between antennal sockets and ocelli nearly impunctate, as well as spaces between lateral ocelli and eyes; inner margin of compound eye with a sparse line of very fine punctures; genal area with sparse scattered punctures; vertex with strong preoccipital carina lined with punctures along margin, a few sparse punctures between lateral ocelli and carina; vestiture sparse, absent from most of face; rear margin of vertex and genae with a fringe of medium-length erect white hairs; similar hairs present between antennal bases, between ocelli, and on mandibles and labrum; a few much shorter hairs on clypeus, along inner eye margins, and on scape.

***Mesosoma***: Scutum moderately punctate in anterior third (1–3 diameters apart) but becoming largely impunctate medially, with just a few punctures posterior of tegulae along lateral and posterior margins (Fig. 3H); scutellum moderately punctured along margins, becoming sparse centrally; metanotum densely but very shallowly punctured; propodeum with dorsal area imbricate to shagreened; propleuron densely punctured; mesopleuron moderately punctured with spaces of 1–3 pit diameters, hypoepimeral area impunctate (Fig. 3J); metapleuron and lower sides of propodeum moderately to densely punctured; fore and mid femora weakly expanded medially and slightly flattened below; fore and mid tibiae slightly narrower than femora, with curved apical spine; hind femur not expanded, of equal width to hind tibia, basitibial plate indistinct; vestiture sparse dorsally; scutum and scutellum hairless except around edges; metanotum with some long white hairs laterally; propleuron with evenly spaced semi-erect medium-length hairs; mesopleuron with long but sparse white hairs, becoming denser on ventral surface; upper sides of propodeum around spiracle with dense patches of short white hairs; fore and mid legs with a few long white hairs on femur and tibia (slightly denser on the latter), scopal hairs on hind leg longer but not much denser; all three basitarsi with denser and more yellow-brown vestiture in comparison to more basal segments of legs.

***Metasoma***: Graduli present on T2–5 and S2–5 (Fig. 3D). T1 smooth with very sparse shallow punctures; T2 pregradular area finely strigulate with few punctures, postgradular area with basal band of dense punctures (~1 diameter apart), narrow transverse line of pits in apical depressed area; T3 pregradular area weakly strigulate with narrow line of punctures, postgradular area densely punctured (0.5–1 diameters), though separated by a smooth region from densely punctured apical depressed area; T4 pregradular area strigulate and punctured apically, postgradular area very densely punctured, pits continuing partway into apical depression which is less sharply defined than on T2 and T3; T5 pregradular area faintly strigulate and densely punctured, postgradular area densely punctured with minute pits continuing partway into apical depression; T6 weakly rugoso-punctate with pits somewhat indistinct; S1 smooth; S2–5 punctures moderate to dense in pregradular areas, dense in postgradular areas; S6 punctures dense but indistinct; vestiture sparse on terga, mostly consisting of short light hairs along graduli, though T5 and T6 with denser mix of light and dark hairs; S2–5 with sparse rows of long, erect, white hairs apically, more uniformly distributed on S6; S2 and S3 additionally with conspicuous, dense patches of appressed white hairs immediately posterior to graduli (wax plates).

##### Etymology.

Named in reference to the type locality, Doi Phu Kha National Park (Nan Province, Thailand).

##### Distribution.

Thailand. Specimens collected from tropical and subtropical moist broadleaf forest biome.

##### Remarks.

Male unknown.

#### Ceratina (Scuticeratina) muscatella

Taxon classificationAnimaliaHymenopteraApidae

Nurse, 1902 stat. rev.

F61E4867-08BC-5632-B167-3E5FFD2F9AD8

Fig. 5A, B

##### Material examined.

India • 2 ♀; “Uttar Pradesh, Bhatta 4 km S Mussoorie”; 1800–2000 m; L. Packer leg.; 6 May 1990; RCYU.

##### Diagnosis.

Distinguished from C. (Scuticeratina) splendida by its lighter reddish-brown abdomen and narrower hind femur (equal in width to hind tibia). Distinguished from C. (Scuticeratina) ferruginea and phukhaensis by the presence of pale maculations on the clypeus (females) and scutellum (both sexes?).

##### Redescription.

**Female (Fig. 5A, B)**. ♀: body length 6.5 mm; forewing 5 mm.

***Color***: Head and mesosoma shiny black; mandibles and labrum black; clypeus with rectangular yellow maculation; paraocular area with darker yellow-brown maculation extending slightly past antennal sockets, filling > 1/2 of space between antennal socket and eye; genal area with yellow-brown maculation fully as long as eye; pronotal collar with pale yellow maculations laterally, as well as most of pronotal lobe; tegulae ferruginous and translucent; scutellum with pale yellow maculation, very narrowly interrupted in apical 1/2 (entirely bisected in one specimen); legs dark brown to ferruginous; fore tibia with indistinct light brown maculation covering ~3/4 of its length; mid tibia with small basal spot; hind tibia with basal maculation covering < 1/4 of its length; T1, T2, and T5 ferruginous brown, T3 and T4 lighter red, T6 black; sterna similar though S4 somewhat darker brown; rims of terga somewhat translucent.

***Head***: Labrum with fairly dense punctation; clypeus with few very sparse punctures except for line of punctures on each side of upper lobe; supraclypeus mostly smooth with few punctures along lateral edges, changing to abruptly punctured between antennal sockets; upper frons between antennal sockets and ocelli nearly impunctate, as well as spaces between lateral ocelli and eyes; inner margin of compound eye with a sparse line of very fine punctures; genal area with a few scattered fine punctures; vertex with strong preoccipital carina lined with punctures along margin; vestiture sparse, absent from most of face; rear margin of vertex and genae with a fringe of medium-length erect white hairs; some similar hairs present between antennal bases, between ocelli, and on mandibles and labrum; a few much shorter hairs on clypeus, along inner eye margins, and on scape.

***Mesosoma***: Scutum moderately punctate in anterior third (1–3 diameters apart) but becoming largely impunctate medially, with just a few punctures along lateral and posterior margins; scutellum moderately punctured along margins, becoming sparse centrally; metanotum densely but very shallowly punctured; propodeum with dorsal area imbricate to shagreened; propleuron densely punctured; mesopleuron moderately punctured with spaces of 2–3 puncture widths, hypoepimeral area impunctate; metapleuron and lower sides of propodeum moderately to densely punctured; fore and mid femora weakly expanded medially and slightly flattened below; fore and mid tibiae slightly narrower than femora, with curved apical spine; hind femur not expanded, of equal width to hind tibia, basitibial plate indistinct; vestiture sparse dorsally; scutum and scutellum hairless except around edges; metanotum with some long white hairs laterally; propleuron with evenly spaced semi-erect medium-length hairs; mesopleuron with long but sparse white hairs, becoming denser on ventral surface; lateral faces of propodeum with moderately dense medium-length white hairs; fore and mid legs with a few long white hairs on femur and tibia (slightly denser on the latter), scopal hairs on hind leg longer but not much denser; all three basitarsi with denser and more yellow-brown vestiture in comparison to more basal segments of legs.

***Metasoma***: Graduli present on T2–5 and S2–5; T1 smooth with very sparse shallow punctures; T2 pregradular area finely strigulate and impunctate, postgradular area smooth and sparsely punctured (2–5 diameters apart), narrow transverse line of pits in apical depressed area; T3 pregradular area strigulate and nearly impunctate, postgradular area with moderately dense punctures (1–3 diameters), though impunctate just before apical depression which contains an additional band of fine and dense punctures; T4 postgradular area moderately punctured, pits continuing partway into apical depression which is less sharply defined than on T2 and T3; T5 postgradular area densely punctured (0.5–1 diameters) with pits continuing partway into apical depression; T6 weakly rugoso-punctate with pits somewhat indistinct; S1 smooth; S2–S3 with moderately dense punctures restricted to postgradular area (1–3 diameters); S4–S5 pregradular area strigulate and with a few scattered punctures, postgradular punctation dense (~1 diameter); S6 strigulate basally, weakly sculptured with indistinct punctures over most of segment (in direct comparison, slightly shinier than in *C. splendida*); vestiture sparse on terga, mostly consisting of short light hairs along graduli, although T5 and T6 with denser mix of light and dark hairs; S2–S5 with sparse rows of long, erect, white hairs apically, more uniformly distributed on S6; S2 and S3 additionally with conspicuous, dense patches of appressed white hairs immediately posterior to graduli (wax plates).

##### Remarks.

*Ceratina muscatella* was originally described from North India by Nurse (1902), who did not assign the species to any subgenus. He described the abdomen as “more or less red”, which may imply some degree of color variation, though the two specimens we examined were similar. Hirashima (1971) examined some specimens from Nurse’s collection including a female *C. muscatella* and a male *C. loquata* (also described by Nurse in the same publication), and suggested they may be synonymous. Nurse’s description suggests that males were collected, but does not provide any male characters, so it is unclear whether males of this species are known.

Hirashima noted some similarities between *C. muscatella* and C. (Catoceratina) perforatrix Smith, 1879, including the largely impunctate head, preoccipital carina, and pale paraocular maculations. However, he also identified significant differences between these species, including the lack of yellow maculations on the labrum and abdomen in *C. muscatella*, as well as the presence of graduli on S2–5 (rather than S2–4 as in *C. perforatrix*), and he ultimately chose to leave the subgeneric status of this species as uncertain.

Besides those listed by Hirashima (1971), additional characters separating *C. muscatella* from *Catoceratina* include the absence of pale maculations on the labrum, frons, and vertex (present in at least some female *C. perforatrix*), presence of maculations on the pronotal lobe and bases of the tibiae (entirely black in *C. perforatrix*, though a stripe is present on the fore femur), translucent ferruginous tegulae (black in *C. perforatrix*), sparser punctation on clypeus and paraocular area, wide impunctate areas on rims of terga (pits reaching apical edge in *C. perforatrix*), indistinct basitibial plate (well-defined in *C. perforatrix*), and absence of ventral scopae (present in *C. perforatrix*). Based on the significant differences between *C. muscatella* and *Catoceratina* and the genetic and morphological similarities with the species of *Scuticeratina* newly described herein, we include *C. muscatella* in the new subgenus as well.

#### Ceratina (Scuticeratina) splendida

Taxon classificationAnimaliaHymenopteraApidae

Shiokawa, 2008 stat. rev.

9D60D9F9-7AB7-5EED-BB5C-0EEAC9A7C547

Fig. 5C, D

##### Material examined.

Pakistan • 1 ♀; “Azad Kashmir, Paras Shogran”; 34.642°N, 73.467°E; M. Kafka leg.; 25 May 2019; RCYU.

##### Diagnosis.

Distinguished from C. (Scuticeratina) muscatella by its entirely black abdomen and slightly enlarged hind femur (~1.25× width of hind tibia). Distinguished from C. (Scuticeratina) ferruginea and phukhaensis by the presence of pale maculations on the clypeus (females) and scutellum (both sexes?).

##### Redescription.

**Female (Fig. 5C, D)**. ♀: body length 7.5 mm; forewing 5.5 mm.

***Color***: Head and mesosoma shiny black; mandibles and labrum black; clypeus with rectangular pale yellow maculation; paraocular area with pale maculation extending slightly past antennal sockets, filling > 1/2 of space between antennal socket and eye; genal area with pale maculation fully as long as eye; pronotal collar with faint grayish maculations laterally, as well as posterior 1/2 of pronotal lobe; tegulae translucent brown; scutellum with large but faint grayish maculation, with V-shaped notch missing from anterior edge; legs dark brown; fore tibia with basal pale maculation covering ~1/2 of its length; mid tibia with tiny, barely noticeable basal spot; hind tibia with basal maculation covering < 1/4 of its length; T1 dark brown, T2–T5 black with rims dark brown and slightly translucent, T6 black; sterna dark brown.

***Head***: Labrum with fairly dense punctation; clypeus with few very sparse punctures except for line of punctures on each side of upper lobe; supraclypeus mostly smooth with few punctures along lateral edges, changing to abruptly punctured between antennal sockets; upper frons between antennal sockets and ocelli nearly impunctate, as well as spaces between lateral ocelli and eyes; inner margin of compound eye with a sparse line of very fine punctures; genal area with a few scattered fine punctures; vertex with strong preoccipital carina lined with punctures along margin vestiture sparse, absent from most of face; rear margin of vertex and genae with a fringe of medium-length erect white hairs; some similar hairs present between antennal bases, between and to both sides of ocelli, and on mandibles and labrum; a few much shorter hairs on clypeus, along inner eye margins, and on scape.

***Mesosoma***: Scutum moderately punctate in anterior quarter (1–3 diameters apart) but becoming largely impunctate medially, with just a few punctures along lateral and posterior margins; scutellum moderately punctured along margins, becoming sparse centrally; metanotum densely but very shallowly punctured; propodeum with dorsal area imbricate to shagreened; propleuron densely punctured; mesopleuron moderately punctured with spaces of 2–3 puncture widths, hypoepimeral area impunctate; metapleuron and lower sides of propodeum moderately to densely punctured; fore and mid femora weakly expanded medially and slightly flattened below; fore and mid tibiae slightly narrower than femora, with curved apical spine; hind femur similarly expanded, ~1.25× wider than hind tibia, basitibial plate indistinct; vestiture sparse dorsally; scutum and scutellum hairless except around edges; metanotum with some long white hairs laterally; propleuron with evenly spaced semi-erect medium-length hairs; mesopleuron with long but sparse white hairs, becoming denser on ventral surface; lateral faces of propodeum with moderately dense medium-length white hairs; fore and mid legs with a few long white hairs on femur and tibia (slightly denser on the latter), scopal hairs on hind leg longer but not much denser; all three basitarsi with denser and more yellow-brown vestiture in comparison to more basal segments of legs.

***Metasoma***: Graduli present on T2–T5 and S2–S5; T1 smooth with very sparse shallow punctures; T2 pregradular area finely strigulate and impunctate, postgradular area smooth and sparsely punctured (2–5 diameters apart), narrow transverse line of pits in apical depressed area; T3 pregradular area strigulate and nearly impunctate, postgradular area with moderately dense punctures (1–3 diameters), though impunctate just before apical depression which contains an additional band of fine and dense punctures; T4 postgradular area moderately punctured, pits continuing partway into apical depression which is less sharply defined than on T2 and T3; T5 postgradular area densely punctured (0.5–1 diameters) with pits continuing partway into apical depression; T6 weakly rugoso-punctate with pits somewhat indistinct; S1 smooth; S2 with moderately dense punctures restricted to postgradular area (1–3 diameters); S3–S5 pregradular area strigulate and with scattered punctures, postgradular punctation dense (~1 diameter); S6 strigulate basally, moderately sculptured with indistinct punctures over most of segment (in direct comparison, slightly duller than in *C. muscatella*); vestiture sparse on terga, mostly consisting of short light hairs along graduli, though T5 and T6 with denser mix of light and dark hairs; S2–S5 with sparse rows of long, erect, white hairs apically, more uniformly distributed on S6; S2 and S3 additionally with patches of appressed white hairs immediately posterior to graduli (wax plates), less conspicuous than in *C. muscatella* but likely just due to wear.

##### Remarks.

*Ceratina splendida* was described from North India, Pakistan, and Nepal by Shiokawa (2008); no male specimens are known. Shiokawa assigned this species to subgenus *Catoceratina* based on the largely impunctate head and preoccipital carina, but distinguished it from *C. perforatrix* based on differences in the clypeal and supraclypeal structure as well as sparser punctation on the mesepisternum.

Shortly after its description however, Warrit (2009) pointed out that *C. splendida* lacks the distinctive ventral scopa seen in *C. perforatrix*, and further showed that the preoccipital carina and largely impunctate face alone are not sufficient to distinguish among several other subgenera. Since it also did not agree well with any other existing subgenus, Warrit tentatively moved *C. splendida* from *Catoceratina* to *Ceratina* s.s., though suggested that it could potentially be placed in a new subgenus with *C. parvula* Smith, 1854 (which itself was split from the nominate subgenus and became the type species of subgenus *Dalyatina* Terzo, 2007).

In addition to the features identified by Shiokawa (2008) and Warrit (2009), all differences between *C. muscatella* and *C. perforatrix* identified above are also applicable for *C. splendida*. Given that *C. splendida* is clearly distinct from *Catoceratina* and that genetic evidence shows a distant relationship to *C. parvula* and *Dalyatina* (Suppl. material 2), we include it with the morphologically similar and phylogenetically close species in subgenus *Scuticeratina*.

### Key to the species of *Scuticeratina* subgen. nov.

At present, we have only been able to examine male specimens of *C. ferruginea*, and so this provisional key may be amended in future with additional material for the other species.

**Table:** 

1	Female with pale maculations on clypeus and scutellum	**2**
–	Female with clypeus and scutellum black	**3**
2	Metasoma black; hind femur ~1.2× wider than hind tibia	**Ceratina (Scuticeratina) splendida**
–	Metasoma partly red; hind femur and tibia of similar width	**Ceratina (Scuticeratina) muscatella**
3	Metasoma entirely black; gena and fore tibia with small, spot-like pale maculations; T2 with dense punctation (pits ~1 diameter apart in pregradular area)	**Ceratina (Scuticeratina) phukhaensis**
–	Metasoma at least partly red, pale maculations on gena and foretibia more extensive, T2 punctation sparser (pits 2–5 diameters apart in pregradular area)	**Ceratina (Scuticeratina) ferruginea**

#### Ceratina (Ceratina) sirikitae

Taxon classificationAnimaliaHymenopteraApidae

Sless, Tucker & Rehan sp. nov.

97C857BC-7D5C-5894-9DD6-BEBFC1B7F537

https://zoobank.org/1B5AD5FD-63A7-4472-BD8E-2B5B15D8F6A0

Fig. 6

##### Type material.

***Holotype***: Thailand • 1 ♀; “Chiang Mai Prov., Alt Queen Sirkit [sic] Botanic Gardens”; 18°52'50.7"N, 98°51'42.3"E; 28 Mar.–3 Apr. 2009; [malaise trap]; Kaewjanta & Sawkord leg.; QSBG-2009-64M; RCYU “rsTha03”. ***Paratypes***: Thailand • 2 ♀; “Chiang Mai Prov., Alt Queen Sirkit [sic] Botanic Gardens”; 18°52'57.5"N, 98°51'39.5"E; 841 m; 20–27 Mar. 2009; [malaise trap]; Kaewjanta & Sawkord leg.; QSGB-2009-50O, QSGB-2009-50D; RCYU “rs2Tha01”, “rs2Tha02” • 1 ♀; “Trang, Nayong, Khaochong”; 7°33.038'N, 99°47.369'E; 75 m; 10-24 Feb. 2005; RCYU “rs2Th05”.

**Figure 6.**
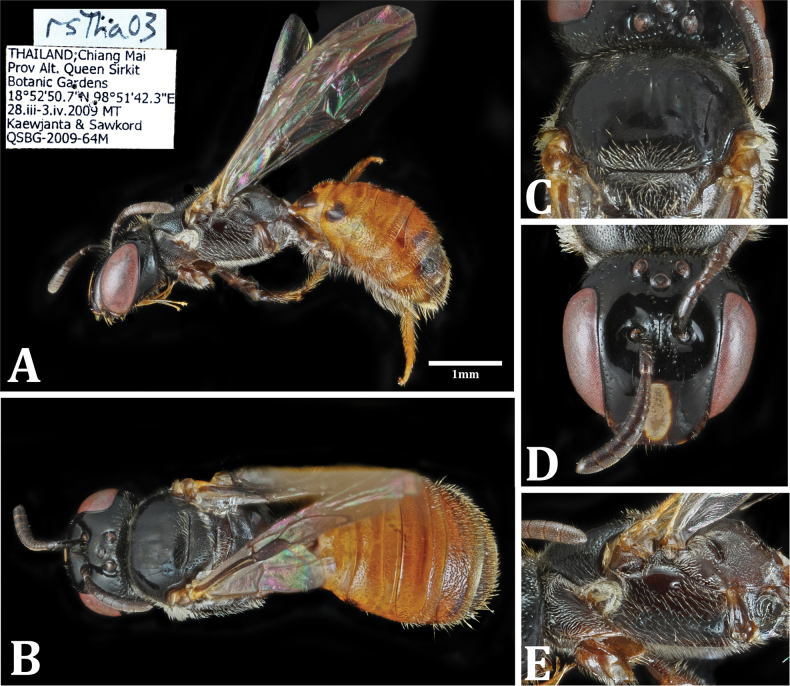
Holotype of Ceratina (Neoceratina) sirikitae sp. nov. **A**. Lateral habitus; **B**. Dorsal habitus; **C**. Mesoscutum; **D**. Face; **E**. Mesopleuron.

##### Diagnosis.

Distinguished from other members of *Ceratina* s.s. (as well as *Neoceratina*) by its red abdomen, foretibia with more extensive maculation, and 2^nd^ submarginal cell nearly closed anteriorly with rs vein absent or extremely narrow.

##### Description.

**Female (Fig. 6)**. ♀ (holotype and paratypes): body length 5–5.5 mm; forewing 3.6 mm; head 1.4 mm wide × 1.4 mm long.

***Color***: Head and mesosoma shiny black (Fig. 6A, B); mandibles ferruginous medially; labrum black; clypeus with longitudinal pale yellow maculation reaching upper margin (Fig. 6D); paraocular area and gena without maculations; pronotal lobes with pale maculation in posterior 1/2; tegulae ferruginous and translucent (Fig. 6C); fore tibia with basal pale spot as well as second, disconnected maculation along ~1/2 of its length (resembling an inverted exclamation mark); mid tibia with tiny, barely noticeable basal spot; hind tibia with maculation covering ~1/3 of its length; tarsi ferruginous; abdomen largely bright red except T1 and S1 dark brown basally, T2 with two dark brown patches at lateral sides, T4 with interrupted dark brown band, T5–T6 dark brown; rims of terga somewhat translucent.

***Head***: Labrum and clypeus sparsely punctured (Fig. 6D); supraclypeus mostly smooth with few punctures along lateral edges, changing to abruptly punctured between antennal sockets; upper frons with two rows of punctures extending above antennal sockets and running between lateral and median ocelli; inner margin of compound eye with a sparse line of fine punctures; dense punctures posterior to ocelli and along preoccipital margin, extending laterally into upper genal area which is moderately punctured; vestiture sparse, absent from most of face; rear margin of vertex and genae with a fringe of medium-length erect white hairs; some similar hairs present between antennal bases, around ocelli, and on mandibles and labrum; a few much shorter hairs on clypeus, along inner eye margins, and on scape.

***Mesosoma***: Scutum moderately punctate in anterior third (1–3 diameters apart), becoming largely impunctate medially except for dense punctures along lateral margins and in posterior sixth of segment (Fig. 6C); scutellum densely punctured throughout (~1 diameter); metanotum densely but shallowly punctured; propodeum with dorsal area imbricate to shagreened; propleuron densely punctured; mesopleuron moderately punctured, with spaces of 1–3 puncture widths, hypoepimeral area mostly impunctate (Fig. 6E); metapleuron densely punctured with minute pits femora weakly expanded medially and slightly flattened below; tibiae slightly narrower than femora, with curved apical spine on fore and mid tibiae, basitibial plate indistinct vestiture sparse dorsally; scutum hairless except around edges; scutellum and metanotum with white hairs over much of surface; propleuron with evenly spaced semi-erect medium-length hairs; mesopleuron with long but sparse white hairs, becoming denser on ventral surface; lateral faces of propodeum with very short but moderately dense white hairs; fore and mid legs with a few long white to light brown hairs on femur and tibia, scopal hairs on hind leg longer but not much denser; all three basitarsi with denser and slightly darker vestiture in comparison to more basal segments of legs.

***Metasoma***: Graduli present on T2–5 and S2–5 (Fig. 6B); T1 smooth; T2 pregradular area weakly strigulate and impunctate, postgradular area moderately punctured (1–3 diameters apart); T3 pregradular area smooth, postgradular area densely punctured (~1 diameter), though impunctate just before densely punctate apical depression; T4–5 postgradular area densely punctured including most of apical depression; T6 densely punctured; S1 smooth; S2–3 postgradular area sparsely punctate (2–5 diameters); S4–S5 postgradular area more densely punctate (1–3 diameters); S6 punctures dense (~1 diameter); vestiture sparse on terga, mostly consisting of short light hairs within apical depressions, though T4–T6 with denser hairs; S2–S5 with sparse rows of long, erect, white hairs apically, more uniformly distributed on S6.

##### Etymology.

Named in reference to the type locality, Queen Sirikit Botanic Garden (Chiang Mai Province, Thailand).

##### Distribution.

Thailand. Specimens collected from tropical and subtropical moist/dry broadleaf forest biomes.

##### Remarks.

Male unknown. We have compared specimens of *C. sirikitae* with other Indomalayan/Eastern Palearctic members of *Ceratina* s.s. recovered as the sister group to *Neoceratina* in the phylogeny of Sless et al. (2024), namely *C. belliata*, *C. iwatai*, *C. sauteri*, and *C. yamanei*. As mentioned in the diagnosis, *C. sirikitae* is easily distinguished from these four species based on the abdominal coloration, though it also has a larger maculation covering roughly 1/2 of the fore tibia, compared to a tiny basal spot or entirely black tibia in the others. As discussed below, this group of species is not closely related to other members of *Ceratina* s.s., and in future should either be united with *Neoceratina* or designated as another new subgenus.

To our knowledge, the red abdomen of *C. sirikitae* is unique among members of both *Ceratina* s.s. and *Neoceratina*, though of course is present in some species of *Scuticeratina* as described above as well as a few species of *Xanthoceratina*, *Calloceratina*, and *Ceratinula* (Michener 2007). However, *C. sirikitae* can easily be distinguished from *Scuticeratina* and *Xanthoceratina* by the absence of paraocular maculations, and presence of punctures (though quite sparse) between antennal bases and ocelli. This species is unlikely to be confused with *Calloceratina*, which is Neotropical in distribution and typically brightly metallic.

*Ceratina sirikitae* is morphologically very similar to some *Ceratinula* despite their significant phylogenetic distance (Fig. 2, Suppl. material 2), including the apically narrowed 2^nd^ submarginal cell (though this feature is also present in various other small-bodied *Ceratina*; Michener 2007), and may run to this subgenus in the key. However, *Ceratinula* is a Neotropical subgenus sharing no range overlap with the Indomalayan *C. sirikitae*, and is included in the Eastern Hemisphere key only due to the presence of *C. arizonensis* in Hawaii where it has been introduced (Hirashima 1971; Michener 2007).

## Discussion and conclusions

Herein we describe three new species of *Ceratina* from Southeast Asia, two of which (*C. ferruginea* and *C. phukhaensis*) are placed into the new subgenus *Scuticeratina*. Two previously described species of uncertain subgeneric status, *C. muscatella* Nurse, 1902 and *C. splendida* Shiokawa, 2008, are also included within *Scuticeratina* on the basis of their genetic and morphological similarities to the newly described species. This work expands the known diversity of small carpenter bees, and further exemplifies the importance of tropical regions for this genus as sources of diversity as noted in previous work (Sless et al. 2024). Our findings also suggest the need for further research on existing species to resolve a number of outstanding taxonomic issues. In our phylogenetic analyses, some members of *Lioceratina* are recovered as the sister group to *Scuticeratina*, though this subgenus is not recovered as monophyletic and requires revision.

Previous work on *C. muscatella* and *C. splendida* has noted some similarities to the subgenus *Catoceratina* Vecht, 1952 (Hirashima 1971; Shiokawa 2008). Lamentably, molecular data from *C. perforatrix* Smith, 1879, the only representative of *Catoceratina*, was not available for inclusion in our phylogeny. As such, it remains to be seen where *C. perforatrix* would fall within the tree and whether it may in fact be closely related to *Scuticeratina*. The ventral scopa described for *C. perforatrix* is a highly unique feature, known only from one other species (the Afrotropical C. (Megaceratina) sculpturata Smith, 1854; Michener 2007; Warrit 2009). Due to the uniqueness of this feature, it seems justified for *Catoceratina* to retain its original monotypic status including only *C. perforatrix*.

The placement of C. (Ceratina) sirikitae sp. nov. also presents broader taxonomic implications for the small carpenter bees. In both our three-gene and larger UCE phylogenies, we recover it within a clade of species currently considered members of *Ceratinasensu stricto* but forming the sister group to *Neoceratina*. Specifically, *C. sirikitae* is sister to C. (Ceratina) belliata Shiokawa, 2008, with C. (C.) iwatai Yasumatsu, 1936, C. (C.) sauteri Strand, 1913, and C. (C.) yamanei Sung & Shiokawa, 2012 forming the rest of this group. The similarities between *Neoceratina* and some members of *Ceratinasensu stricto* have previously been noted by Hirashima (1971) and Michener (2007), the latter of whom suggested that these subgenera grade into each other. Females of these subgenera are only distinguished by the presence of a gradulus on S5 in Michener’s key, which can be difficult to evaluate in some specimens. Furthermore, the broader phylogeny reveals that *Ceratina* s.s. is highly polyphyletic, with members of this subgenus scattered throughout the tree and often distantly related to the type species *Ceratina cucurbitina* (Rossi, 1792) (Sless et al. 2024). Though beyond the scope of this paper, we suggest that a revision of *Neoceratina* is necessary and that these phylogenetically related members of *Ceratina* s.s., as well as the newly described *C. sirikitae*, should either be included within *Neoceratina* or raised as a new subgenus.

## Supplementary Material

XML Treatment for Ceratina (Scuticeratina)

XML Treatment for Ceratina (Scuticeratina) ferruginea

XML Treatment for Ceratina (Scuticeratina) phukhaensis

XML Treatment for Ceratina (Scuticeratina) muscatella

XML Treatment for Ceratina (Scuticeratina) splendida

XML Treatment for Ceratina (Ceratina) sirikitae

## Additional information

### Use of AI

No use of AI was reported.

### Author ORCIDs

Trevor J. L. Sless https://orcid.org/0000-0002-5833-3049

### Data availability

All of the data that support the findings of this study are available in the main text or Supplementary Information.

## Supplementary materials

Supplementary material 1 Full three-gene phylogeny including all specimens and outgroups Data type png Explanation note Support values at nodes represent ultrafast bootstrap and SH-alrt values, respectively. Newly described species of subgenus *Scuticeratina* are in blue and the new species C. (Ceratina) sirikitae is in green. https://binary.pensoft.net/file/1539709This dataset is made available under the Open Database License (http://opendatacommons.org/licenses/odbl/1.0/). The Open Database License (ODbL) is a license agreement intended to allow users to freely share, modify, and use this Dataset while maintaining this same freedom for others, provided that the original source and author(s) are credited. Trevor J. L. Sless, Erika M. Tucker, Sandra M. Rehan

Supplementary material 2 Full UCE phylogeny including all specimens and outgroups Data type png Explanation note Support values at nodes represent ultrafast bootstrap and SH-alrt values, respectively. The new species Ceratina (Scuticeratina) ferruginea and C. (Ceratina) sirikitae are in blue and green, respectively. https://binary.pensoft.net/file/1539710This dataset is made available under the Open Database License (http://opendatacommons.org/licenses/odbl/1.0/). The Open Database License (ODbL) is a license agreement intended to allow users to freely share, modify, and use this Dataset while maintaining this same freedom for others, provided that the original source and author(s) are credited. Trevor J. L. Sless, Erika M. Tucker, Sandra M. Rehan

Supplementary material 3 Additional information Data type xlsx Explanation note **table SS1**. Genetic divergence (% bp difference, Kimura 2-parameter, below diagonal) and absolute number of nucleotide differences (above diagonal) for the COI gene region (1284 bp) within and between *Scuticeratina* and related species of *Lioceratina* and *Xanthoceratina*, as well as *Ceratina sirikitae* and related species of *Ceratina**sensu stricto* and *Neoceratina*. **table S2**. Genetic divergence (% bp difference, Kimura 2-parameter, below diagonal) and absolute number of nucleotide differences (above diagonal) for the CytB gene (429 bp) within and between *Scuticeratina* and related species of *Lioceratina* and *Xanthoceratina*, as well as *Ceratina sirikitae* and related species of *Ceratina**sensu stricto* and *Neoceratina*. https://binary.pensoft.net/file/1539711This dataset is made available under the Open Database License (http://opendatacommons.org/licenses/odbl/1.0/). The Open Database License (ODbL) is a license agreement intended to allow users to freely share, modify, and use this Dataset while maintaining this same freedom for others, provided that the original source and author(s) are credited. Trevor J. L. Sless, Erika M. Tucker, Sandra M. Rehan
