# Supplementary figures and images for: Scuticeratina: A new subgenus of small carpenter bees (Hymenoptera, Apidae, Xylocopinae) from Indomalaya
Source: Zookeys. 2026 Feb 13;1269:151–80. doi: 10.3897/zookeys.1269.148092 (PMC12924047; doi:10.3897/zookeys.1269.148092)

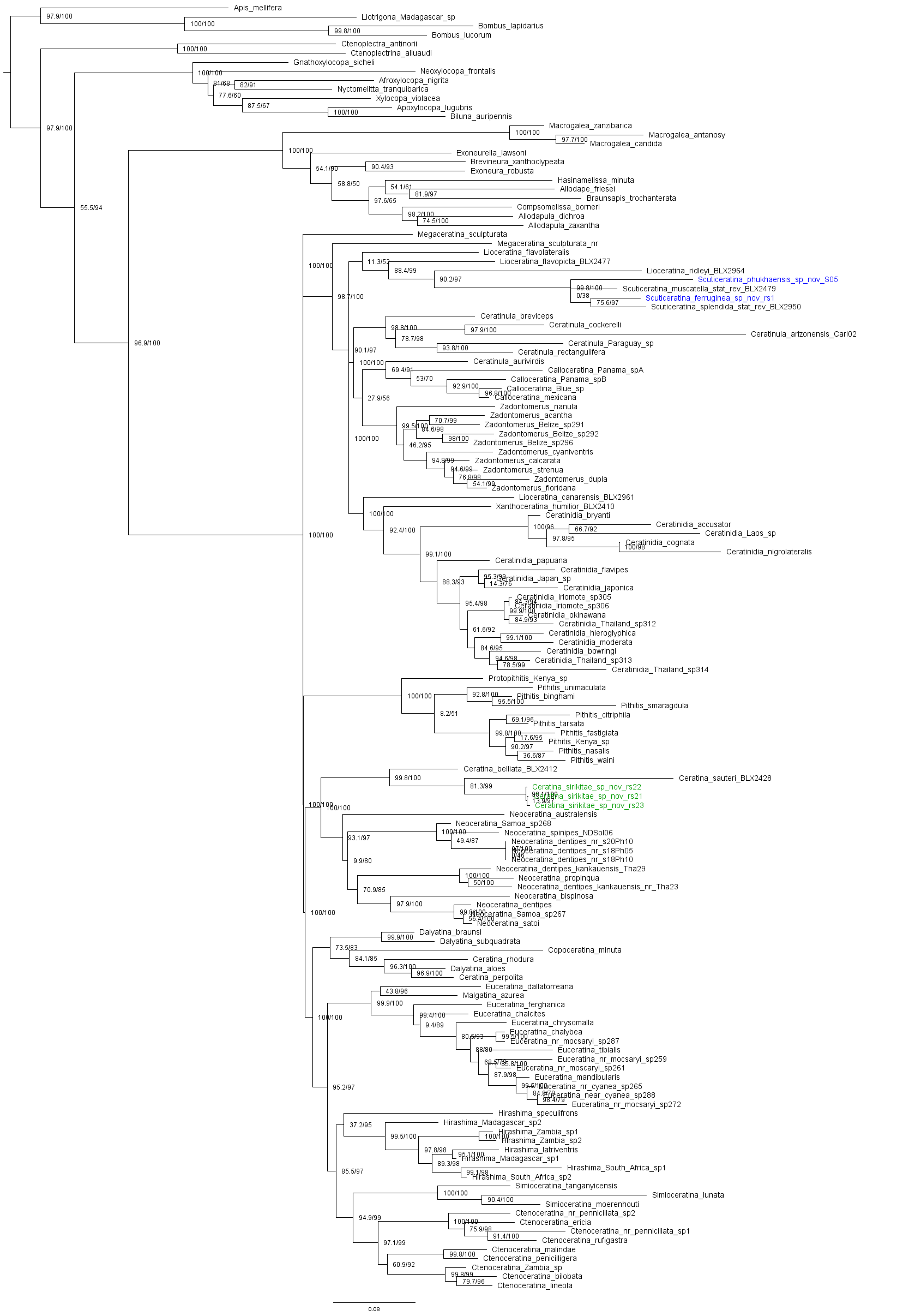

Supplement: Supplementary material 1 — Full three-gene phylogeny including all specimens and outgroups [file zookeys-1269-151_article-148092__-s001.png]

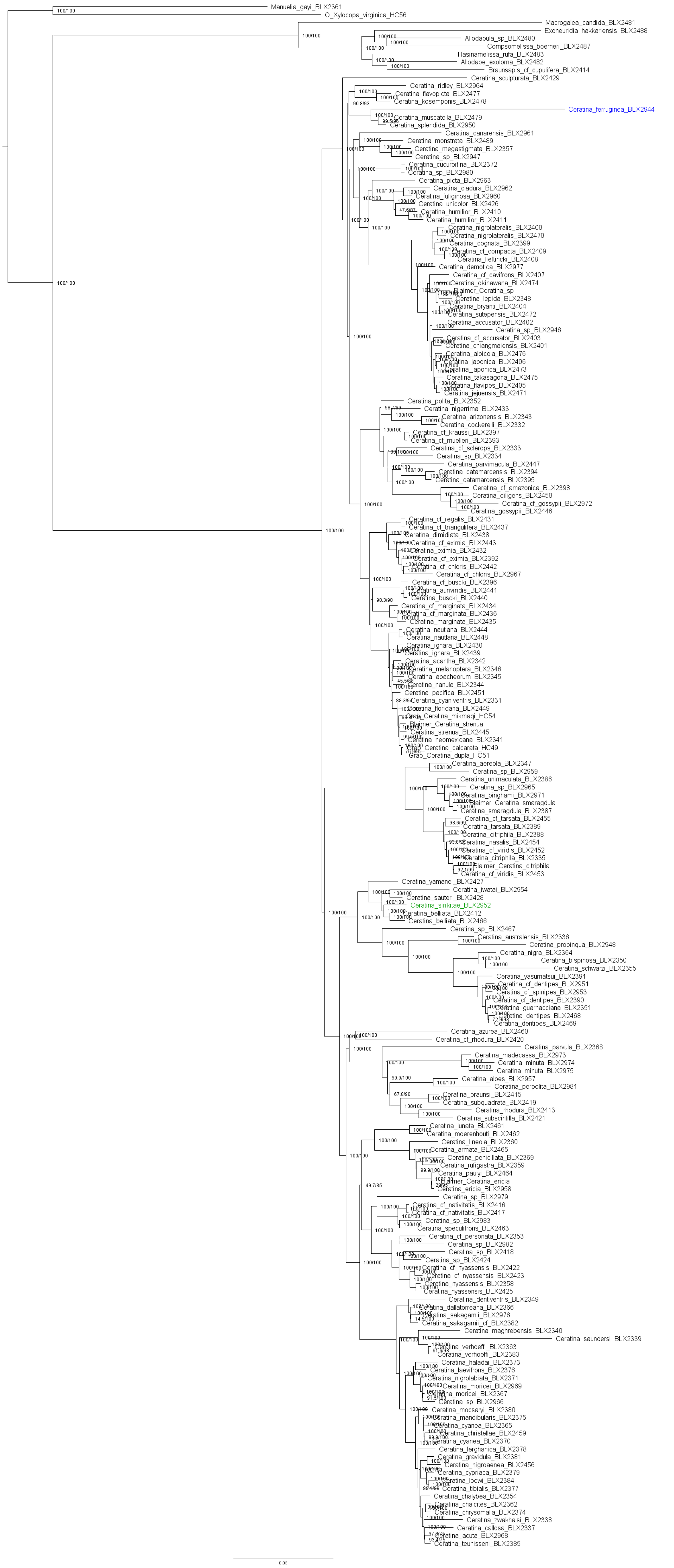

Supplement: Supplementary material 2 — Full UCE phylogeny including all specimens and outgroups [file zookeys-1269-151_article-148092__-s002.png]
